# Analytical and preparative separation of phosphorothioated oligonucleotides: columns and ion-pair reagents

**DOI:** 10.1007/s00216-019-02236-9

**Published:** 2019-12-09

**Authors:** Martin Enmark, Joakim Bagge, Jörgen Samuelsson, Linda Thunberg, Eivor Örnskov, Hanna Leek, Fredrik Limé, Torgny Fornstedt

**Affiliations:** 1 Department of Engineering and Chemical Sciences, Karlstad University, 651 88 Karlstad, Sweden; 2 Pharmacognosy, Department of Medicinal Chemistry, Biomedical Centre, Uppsala University, Box 574, 751 23 Uppsala, Sweden; 3 Early Chemical Development, Pharmaceutical Sciences, BioPharmaceuticals R&D, AstraZeneca, Gothenburg, 431 83 Mölndal, Sweden; 4 Advanced Drug Delivery, Pharmaceutical Sciences, BioPharmaceuticals R&D, AstraZeneca, Gothenburg, 431 83 Mölndal, Sweden; 5 Nouryon, Separation Products, 445 80 Bohus, Sweden

**Keywords:** Ion-pair RPLC, Therapeutic oligonucleotides, Phosphorothioate, Diastereomers, Overloaded peaks, Preparative separations

## Abstract

Oligonucleotide drugs represent an emerging area in the pharmaceutical industry. Solid-phase synthesis generates many structurally closely related impurities, making efficient separation systems for purification and analysis a key challenge during pharmaceutical drug development. To increase the fundamental understanding of the important preparative separation step, mass-overloaded injections of a fully phosphorothioated 16mer, i.e., deoxythymidine oligonucleotide, were performed on a C18 and a phenyl column. The narrowest elution profiles were obtained using the phenyl column, and the 16mer could be collected with high purity and yield on both columns. The most likely contribution to the successful purification was the quantifiable displacement of the early-eluting shortmers on both columns. In addition, the phenyl column displayed better separation of later-eluting impurities, such as the 17mer impurity. The mass-overloaded injections resulted in classical Langmuirian elution profiles on all columns, provided the concentration of the ion-pairing reagent in the eluent was sufficiently high. Two additional column chemistries, C4 and C8, were also investigated in terms of their selectivity and elution profile characteristics for the separation of 5–20mers fully phosphorothioated deoxythymidine oligonucleotides. When using triethylamine as ion-pairing reagent to separate phosphorothioated oligonucleotides, we observed peak broadening caused by the partial separation of diastereomers, predominantly seen on the C4 and C18 columns. When using the ion-pair reagent tributylamine, to suppress diastereomer separation, the greatest selectivity was found using the phenyl column followed by C18. The present results will be useful when designing and optimizing efficient preparative separations of synthetic oligonucleotides.

**Electronic supplementary material:**

The online version of this article (10.1007/s00216-019-02236-9) contains supplementary material, which is available to authorized users.

## Introduction

Therapeutic oligonucleotides (ONs) represent a recent breakthrough in the pharmaceutical industry [1–3]. As of 2019, at least five ONs have entered commercial phase and hundreds are in clinical trials [2]. One important therapeutic class of ONs is the so-called antisense oligonucleotide (ASO) [2, 4, 5].

ASOs are commonly manufactured via β-cyanoethyl phosphoramidite solid-phase synthesis [6]. This method results in many structurally similar impurities. The polymeric nature of the ON and the many impurities challenge analytical and preparative separations [7, 8]. The deletion or addition of one nucleotide in a full-length product of length *n* results in what is typically referred to as a shortmer (*n* − 1) or longmer (*n +* 1) impurity and is directly related to failure in the solid-phase synthesis. The regulatory bodies have not yet published reporting threshold guidelines for ON impurities. However, a position paper addressing ON impurities proposes a reporting threshold of 0.2% for the impurities [8], making research into the chromatographic isolation of full-length product of desired purity and yield from shortmer and longmer impurities highly relevant. From the perspective of both analysis and purification, selectivity for the (*n* − 1) and (*n* + 1) impurities poses a great challenge, becoming increasingly difficult with increasing ON length [9]. ONs are expensive to synthesize compared with small molecules, so the chromatographic methods used for preparative isolation must be optimized for both productivity and yield which due to the non-linearity of “overloaded” elution profiles poses a greater challenge than optimizing analytical chromatography conditions [10].

Reversed-phase liquid chromatography (RPLC) and ion-exchange chromatography are established methods for preparative purification of ONs in gram to kilogram scale [11] either with silica or polystyrene support [6]. Ion-pair reversed-phase liquid chromatography (IP-RPLC) has also been exploited for separation of ONs [12] and has gained in popularity according to a recent review [6]. Even if C18 stationary phases are most common for separation of ONs [7], various other column chemistries have been investigated among other alternative selectivity found by using phenyl-based stationary phases [13, 14].

The use of different ion-pair reagents for a successful analysis in analytical IP-RPLC was recently investigated [7, 15] including tertiary alkylamines such as triethyl-, tributyl-, and tripentylamine, as well as primary and quaternary amine. Fewer data are available regarding purification, but a recent industry review suggests that the use of triethylamine is prevalent [6].

Phosphorothioation is the most common chemical modification of ONs [16, 17]. Much research has been focused on developing chromatographic methods to analyze phosphorothioate (PS)-modified ONs, which was recently summarized in a comprehensive review, including discussion about column chemistry and ion-pairing reagents [7]. A common separation challenge is the partial separation of the diastereomers introduced by the PS modification [7, 18–20]. This leads to peak broadening and increased difficulty in separating structurally very similar impurities. In a previous study [20] of PS-modified ONs, we found that selecting the ion-pair reagent in the eluent was crucial for the diastereomer selectivity and the use of trimethylammonium acetate followed by triethylammonium acetate resulted in the best selectivity. On the other hand, the use of tributyl ammonium acetate in the eluent suppressed diastereomer selectivity [20].

In this study, we started out from the conclusions from the analytical study [20] and now focus on longer oligonucleotides, more similar in size as ASOs, i.e., around 15–25 nucleotides. In order to focus on the study on the effect of peak broadening due to partial diastereomer separation (see above), we will only consider homomeric oligonucleotides whose deletion products (*n* − 1, etc.) have a predictable retention pattern. Therapeutic ASOs consist of mixed nucleobases which when separated will give a more complex retention pattern [9], and although interesting, remain beyond the scope of the current study. The homomeric oligonucleotides used in this study represents realistic key model solutes towards reaching a deeper knowledge of separation mechanisms for therapeutic ASOs.

The research methodology was to use the knowledge achieved in a recent fundamental study [20] for developing improved ion-pair liquid chromatographic phase systems for not only analytical but also preparative separations of longer ONs (as models for ASOs). Four RPLC stationary-phase ligands, i.e., C4, C8, C18, and phenyl, were studied and both native and fully PS-modified deoxythymidine oligonucleotides were used as model compounds. In the analytical part of the study, the peak broadening of PS-modified oligonucleotides caused by partial diastereomer separation was investigated on different columns using triethyl- or tributylammonium acetate ion-pairing reagents. Overloaded peaks were studied using a series of mass-overloaded injections to investigate the column elution profiles. Finally, the most promising columns were investigated further by performing fraction analysis of overloaded injections of a fully phosphorothioated ON 16mer in order to quantify the purity as a function of the cut-point. In addition, quantitative information about the displacement effects of shortmers was obtained.

## Material and methods

### Chemicals

Two ion-pairing reagents triethylammonium acetate (TEtAA) or tributylammonium acetate (TBuAA) were used, prepared from triethylamine (≥ 99.5%) or tributylamine (≥ 99.5%) together with acetic acid (≥ 99.8%); all were purchased from Sigma-Aldrich (St. Louis, MO, USA). Mobile phases were prepared using HPLC grade acetonitrile from VWR (Radnor, PA, USA) and deionized water with a resistivity of 18.2 MΩ cm from a Milli-Q Advantage A10 water purification system from Merck Millipore (Darmstadt, Germany). All ON samples were purchased from Integrated DNA Technologies (Leuven, Belgium). ONs were received in lyophilized form and were used without further purification.

### Columns

Four reversed-phase columns, i.e., C4, C8, C18, and phenyl, were investigated. All columns were 150 × 3.0 mm with a particle size of 2.5 μm and a pore size of 100 Å and were provided by Kromasil (Bohus, Sweden); see Table 1 for more column properties. A Waters ACQUITY UPLC BEH C18 column (particle size 1.7 μm, 100 × 2.1 mm I.D.) was used to analyze fractions. The 1.7 μm BEH C18 column was used with a UHPLC instrumentation (see below) to maximize resolution and thereby achieving more accurate purity profiles compared with using HPLC that is standard at fraction analysis.

**Table 1:** Properties of investigated columns, provided by the manufacturer. All columns were 150 × 3.0 mm and the particle size was 2.5 μm

Column	Ligand density (μmol m^−2^)	Surface area (m^2^ g^−1^)	Pore volume (cm^3^ g^−1^)	Packing density (g cm^−3^)	Total carbon content (w/w %)
C4	3.8	318	0.85	0.57	7.9
C8	3.7	328	0.83	0.60	11.8
C18	3.5	313	0.85	0.66	19.8
Phenyl	3.7	327	0.83	0.59	14.1

All data specified by vendor

### Instrumentation

All experiments except fraction collection and subsequent analysis were conducted on an Agilent 1200 HPLC system (Agilent Technologies, Palo Alto, CA, USA), configured with a binary pump, a 100-μL injection loop, a diode-array UV detector, and a column thermostat. Fraction collection experiments were conducted using a Waters ACQUITY I-Class UPLC system (Waters, Mississauga, ON, Canada) configured with a binary pump, a 100-μL injection loop, a diode-array UV detector, a single quadrupole detector (Waters QDa) operating in ESI negative mode, a column thermostat, and a fraction collector. Fraction analysis was performed on a Waters ACQUITY UHPLC system configured with a binary pump, a 10-μL injection loop, a diode-array UV detector, and a single quadrupole detector (Waters SQD) operating in ESI negative mode. The temperature of the column thermostat was set to 50 °C for all experiments, a typical temperature for minimizing the risk for formation of secondary structures of ONs, although this risk is minimal for the selected model compounds.

### Procedures

All samples were prepared by dissolving the lyophilized samples in deionized water followed by vortexing and heat treatment for about 10 min at 50 °C. The following simplified naming convention was used for the ONs: for example, a 5mer deoxythymidine monophosphate with one sulfur in each phosphodiester linkage, 5′-T_S_T_S_T_S_T_S_T-3′, is referred to as T5. A T5–T20 ladder was prepared by initially dissolving T5, T10, T15, and T20 homomers to a concentration of 0.7 mg mL^−1^ in 2 mL of deionized water. Each homomer was then diluted to a final concentration of 0.1 mg mL^−1^. A sample mixture of T12, T16, and T20 was prepared in a similar way. An additional T17 samples of 0.1 mg mL^−1^ were also prepared. T16 samples were prepared at 0.1, 9.38, and 30 mg mL^−1^. An uracil sample of 0.1 mg mL^−1^ was prepared in deionized water and used as the void volume marker.

All eluents and ion-pairing reagents were prepared by mass. Molar concentrations of the ion-pairing reagent were estimated from the calculated volume of the eluent. While this introduces a slight concentration error as the volumes are not additive, it allows for better reproducibility between eluent preparations. Stirring continued for at least 1 h for TEtAA eluent and at least 12 h for TBuAA eluent to allow complete dissolution, as verified by ocular inspection. Eluents were vacuum degassed for approximately 5–10 min before being used. Columns were equilibrated using at least 50 column volumes when switching between ion pairs and at least 5 column volumes when varying the ion-pair reagent concentration. Analytical injections were recorded at 260 nm and overloaded injections at 280 and 300 nm. All experiments were conducted at 0.42 mL min^−1^, except for the C18 and BEH C18 columns, for which 0.33 and 0.6 mL min^−1^ were used, respectively. In this context, it should be mentioned that the C18 column had a higher back pressure than the other columns; thus, the flow rate had to be reduced and the gradient program was adjusted accordingly to maintain constant slope. The higher back pressure is likely due to the higher packing density of the C18 column (see Table 1).

All gradient programs and ion-pair reagent concentrations used are listed in Table 2.

**Table 2:** Summary with details about gradient methods used in the study

Experiment	Ion pair (mM)	Column	Gradient start (v% MeCN)	Gradient slope (v% mL^−1^)
See Figs. 1, 2, and 3	50 mM TEtAA	C4, C8, C18, and phenyl	10.15	0.47
	5 mM TBuAA		36	0.95
See Fig. 4	10 mM TBuAA	C8	45.6	1.14
	20 mM TBuAA		48.6	
	30 mM TBuAA		49.8	
See Figs. 5, 6, and 7	30 mM TBuAA	C4	51.6	1.14
		C8	51.6	
		C18	51.3	
		Phenyl	49.2	
See Fig. 6 (purity analysis)	10 mM TBuAA	BEH C18	30	1.6

## Results and discussion

The retention behavior of deoxythymidine ONs was evaluated using ion-pair RPLC with two different ion-pair reagents and four different stationary phases. The results are presented in three parts: First, the selectivity and diastereomer separation are evaluated for unmodified and PS-modified ONs using two different ion-pairing reagents on four different columns. Next, the mass overloading of T16 is presented on each column, together with a study of how the peak shape is influenced by the ion-pairing reagent concentration. Finally, the results of fraction analysis of overloaded injections of T16 on C18 and phenyl are presented, including a detailed quantitative description of the elution of the (*n* − 1) through (*n* − 3) and (*n* + 1) impurities.

### Selectivity and diastereomer peak broadening

Most preparative chromatographic method development begins by investigating the selectivity and resolution when using various columns and eluents [21, 22]. Combinations that display high selectivity between target and important impurities are then typically investigated by volume overloading at the solubility limit of the sample. Based on our previous findings that PS-modified short ONs display complete or partial diastereomer separation [20], we wanted to investigate the effect of column chemistry on diastereomer separation for longer ONs.

**Fig. 1:**
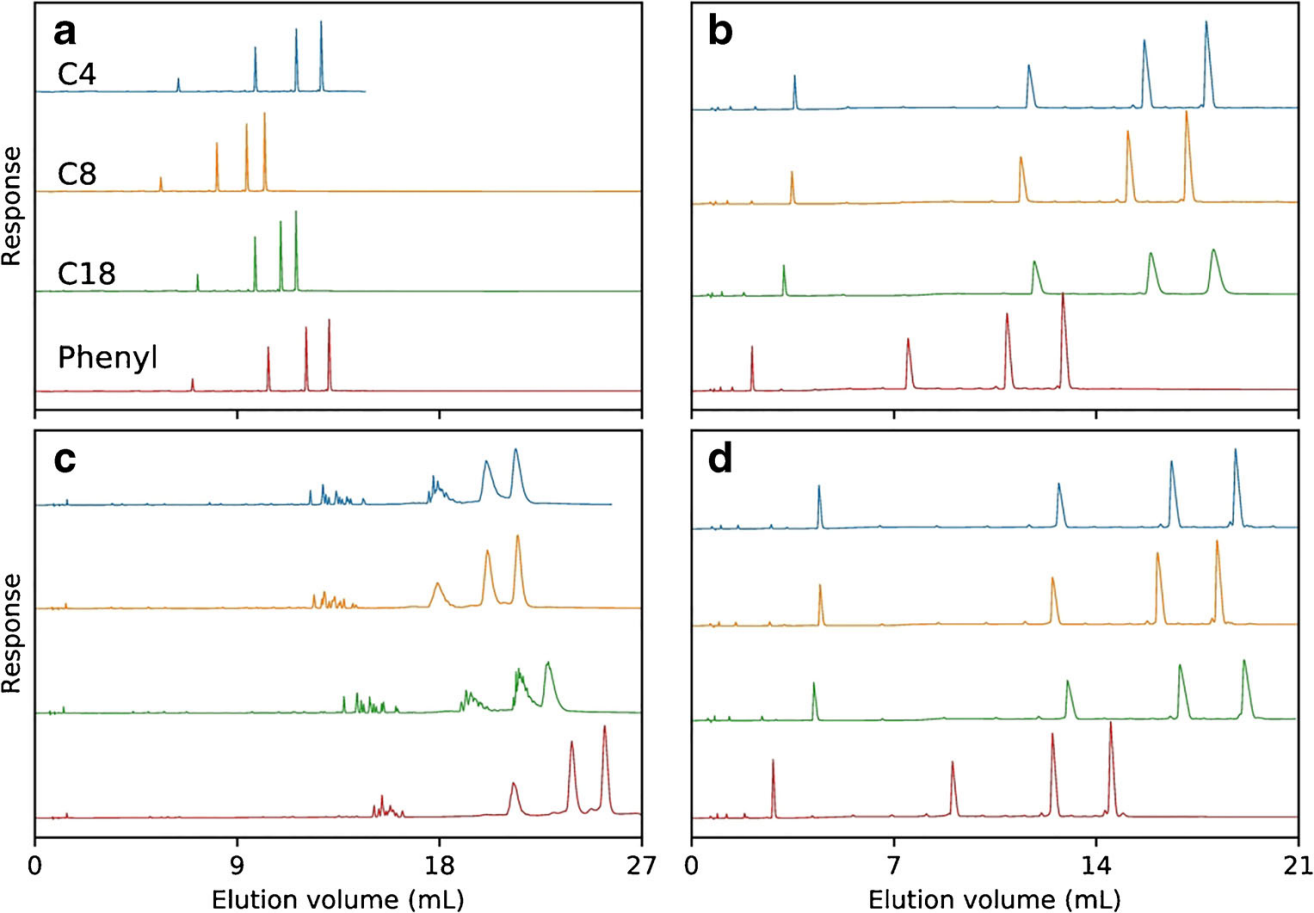
Mixture of T5, T10, T15, and T20, separated on Kromasil C4, C8, C18, and phenyl columns using either triethylammonium acetate (TEtAA) or tributylammonium acetate (TBuAA) as ion-pairing reagent. Plot **a** shows chromatograms using native oligonucleotides eluted in 50 mM TEtAA. **b** The same sample is separated using 5 mM TBuAA. Plots **c** and **d** show the elution profiles of a PS-modified oligonucleotide, under identical conditions as in (**a**) and (**b**), respectively. See Table 2 for method details

**Fig. 2:**
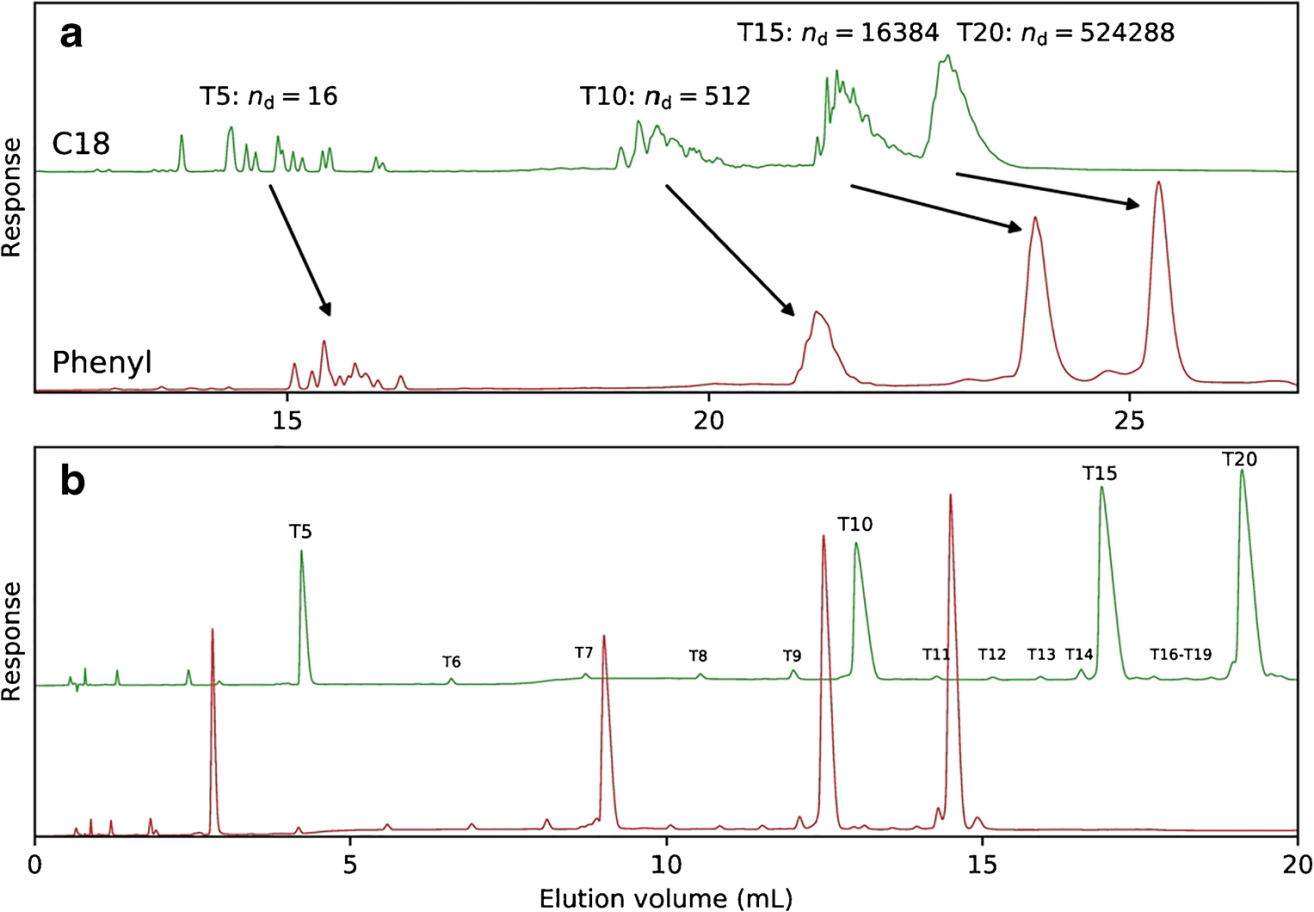
Detailed chromatograms from Fig. 1 for 5-μL injections of PS-modified oligonucleotide sample mixtures, i.e., 0.1 mg mL^−1^ T5, T10, T15, and T20, eluted using either TEtAA (**a**) or TBuAA (**b**) as the ion-pairing reagent. Plot **a** shows the different apparent diastereomer separations obtained using either C18 or phenyl columns when separating the sample under identical conditions, using 50 mM TEtAA. Plot **b** shows the same separation as in **a** but instead using 5 mM TBuAA. The theoretical number of diastereomers for each oligonucleotide is written as *n*_d_. See Table 2 for method details

Two sets of 5-, 10-, 15-, and 20mer deoxythymidine monophosphate ONs, as either native or PS-modified phosphodiesters, were injected. The native ONs display complete resolution when using both TEtAA and TBuAA (Fig. 1a and b), but significantly wider peaks are observed when using TBuAA (Fig. 1b). This might be explained by increased column overload at the lower ionic strength when using TBuAA rather than TEtAA. The order of the retention time for T20 is phenyl > C4 > C18 > C8 when using TEtAA and C18 > C4 > C8 > phenyl when using TBuAA. When the set of fully PS-modified ONs is separated using either ion-pairing reagent, the relative order of the retention time on the different columns is preserved (Fig. 1c and d). When using TEtAA, the effect of complete or partial diastereomer separation leads to significant loss of resolution (Fig. 1c). In Fig. 1d, fully PS-modified ONs are separated using TBuAA and the retention order of T20 is C18 > C4 > C8 > phenyl. These results indicate that the diastereomer separation can be suppressed when using TBuAA. Of all the alkyl ligands, C18 has the highest diastereomer selectivity, whereas the arylic phenyl ligand has the lowest (Fig. 1c). For T5, which has 16 diastereomers, approximately 13 can be partially resolved on the C18 column and approximately 10 on the phenyl column (Fig. 2a). Furthermore, due to the peak broadening caused by high diastereomer selectivity, T15 cannot be baseline resolved from T20. However, on the phenyl column, they can easily be resolved. This marked difference can likely be attributed to stronger hydrophobic interactions with C18, introducing a more pronounced diastereomer separation. With the use of TBuAA, it is possible to achieve an (*n* − 1) resolution up to at least T15 (Fig. 2b). Thus, the selection of TBuAA as ion-pairing reagent is recommended for a given separation system, to reach maximal resolution, by most efficiently suppressing the diastereomer selectivity.

**Fig. 3:**
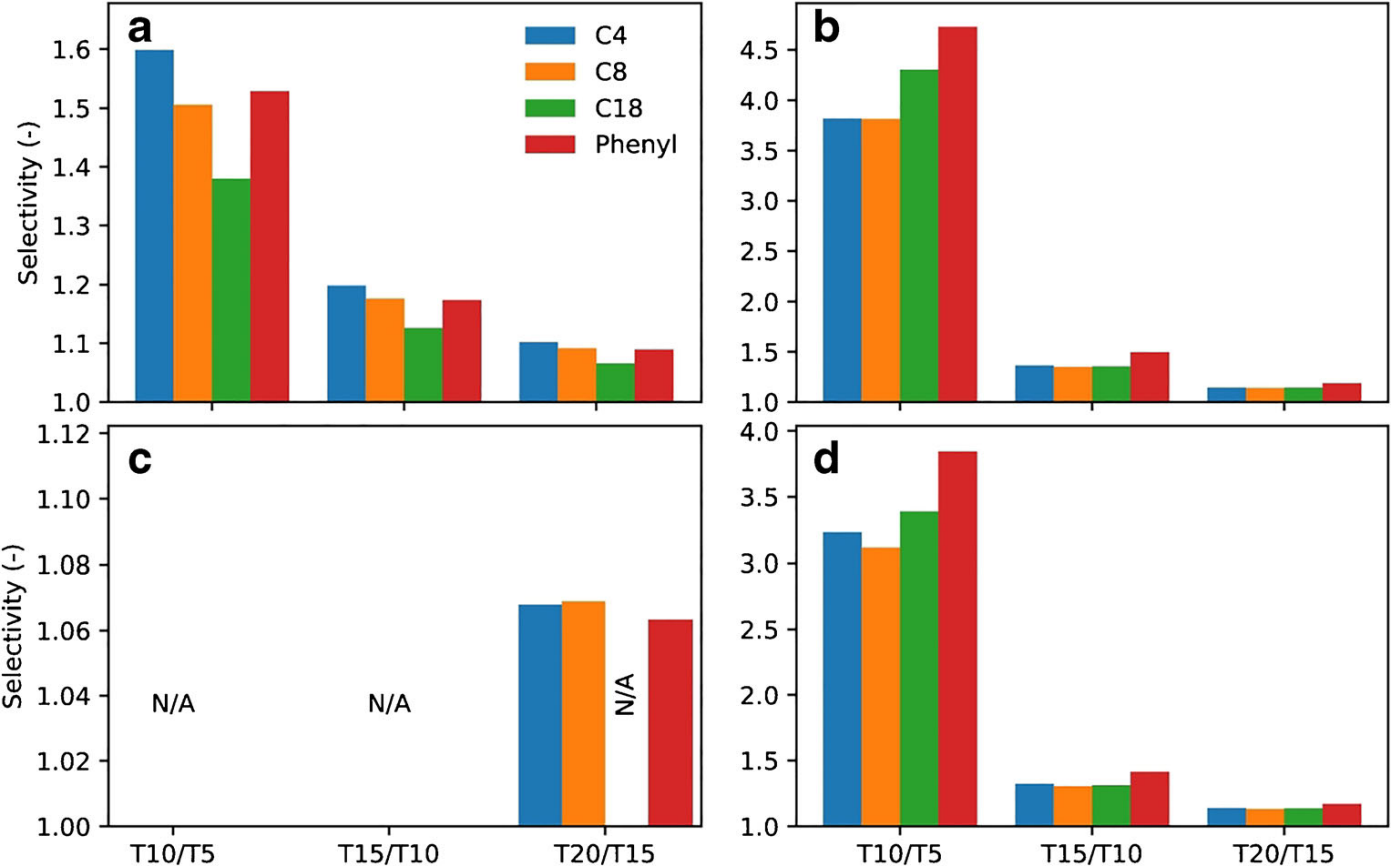
Selectivity between the major peaks of the oligonucleotide samples T5, T10, T15, and T20 eluted on the different columns. The first row (**a**, **b**) summarizes the selectivity when eluting the native oligonucleotides with TEtAA (**a**) or TBuAA (**b**) under identical conditions as in Fig. 1. The second row (**c**, **d**) details the selectivity obtained using PS-modified oligonucleotide samples with TEtAA (**c**) or TBuAA (**d**). Due to significant diastereomer separation, it was impossible to calculate the selectivity between any oligonucleotides using the C18 column and TEtAA but only when using the phenyl, C4, and C8 columns and then only between T20 and T15 (**c**)

When separating native ONs using TEtAA, the selectivity across the investigated ON length decreases with increasing length and is ranked as C4 > phenyl > C8 > C18 (Fig. 3a). For PS-modified ONs, the selectivity can only be calculated between T20 and T15 and only for the C4, C8, and phenyl columns because of the diastereomeric selectivity for short ONs (Figs. 1c and 3c). For native ONs separated using TBuAA, the column selectivity is ranked as phenyl > C18 > C8 ≈ C4, but this difference is diminishing with increasing length of the ON. In the case of modified ONs, the ranking is similar, and the main difference is a decrease in absolute selectivity. Notably, the absolute selectivity between T10 and T5 is about three times higher when using TBuAA rather than TEtAA even though the separation time for TBuAA is shorter.

The ion-pairing reagent of TBuAA suppressed diastereomer resolution and should be preferred over TEtAA since the most recent generation of ASOs typically contains PS modifications. Maximum length-based selectivity will then be obtained using the phenyl column followed by C18. All further experiments were therefore performed using TBuAA and fully PS-modified ONs.

In the final product, as example in the case of therapeutic ASOs, the counter ion must be exchanged after the elution of the chromatographic fraction, commonly to a sodium ion or to an ammonium ion [6]. Hence, the choice of ion-pair in the mobile phase has no influence on the quality of the final product. However, the difficulty of removal of the ion-pair reagent from the final product depends on the type reagent used in the separation. However, this is not the scope of this study.

### Mass-overloaded injections of PS-modified T16

**Fig. 4:**
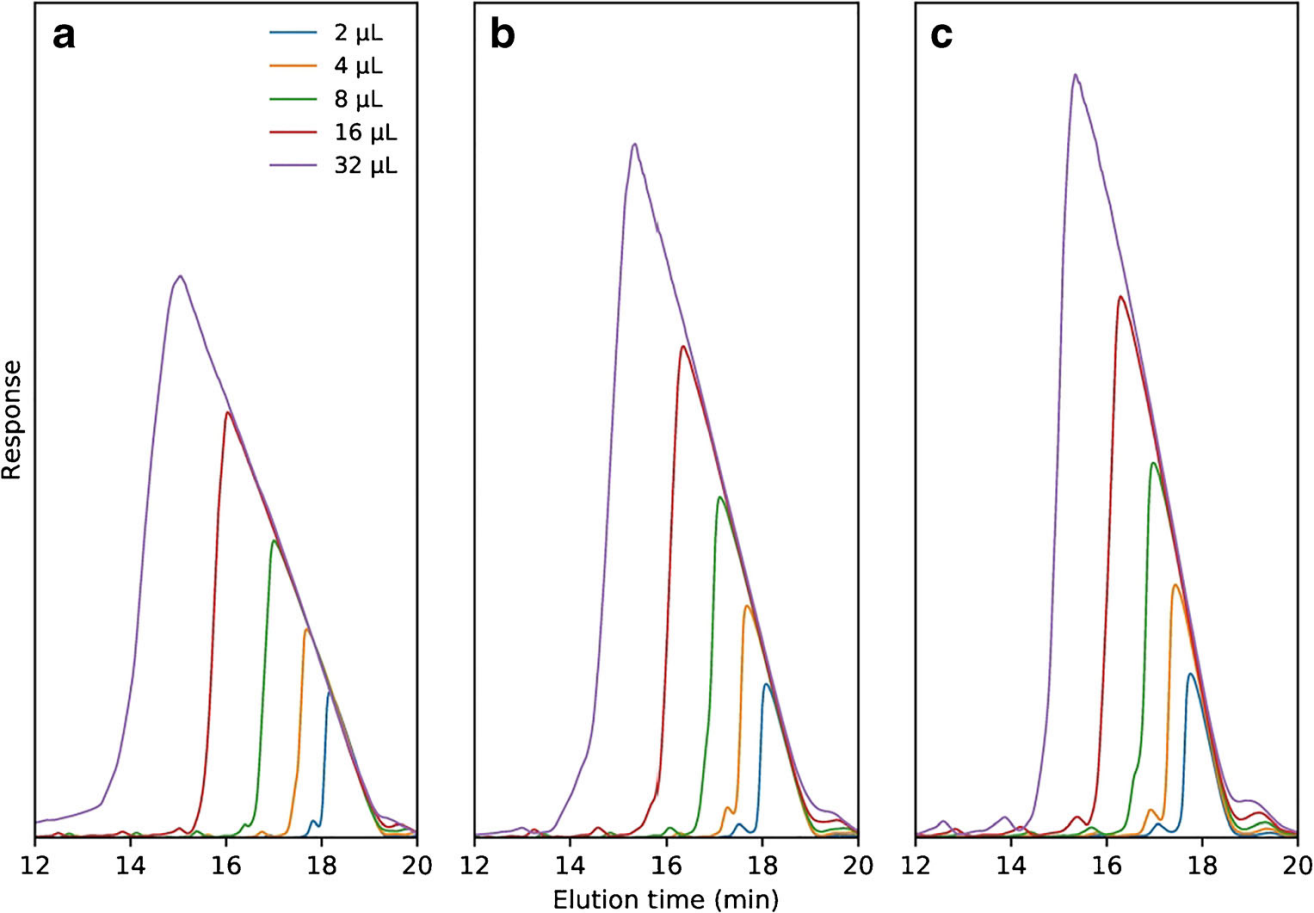
Chromatograms of preparative injections of 2–32 μL of 9.38 mg mL^−1^ PS-modified T16 on the Kromasil C8 column with 10, 20, and 30 mM TBuAA (**a**–**c**). Gradient start adjusted to normalize the retention volume at each level. See Table 2 for method details

Based on the observation that TBuAA is the preferred ion-pairing reagent to suppress diastereomer selectivity and also to increase resolution between the (*n* − 1) shortmers, it is relevant to investigate the impact of TBuAA concentration. If we assume a one-to-one stoichiometric charge ratio of the TBuAA ion-pairing reagent and the solute, the concentration must be increased linearly with the length of the ONs. In the case of a 16mer ON (T16), with a formal charge of − 15 at pH 8, the concentration of TBuAA should be at least 15 times that of T16. A sample with the concentration 9.68 mg mL^−1^ (1.9 mM) was injected on a C8 column in five volumes, between 2 and 32 μL. TBuAA concentrations of 10, 20, and 30 mM were evaluated, corresponding to approximately 30, 60, and 100% stoichiometric ratios to the 1.9 mM sample evaluated (Fig. 4). The gradient slope was 1.14 v% min^−1^ under all conditions, but the starting point was adjusted to obtain an identical analytical retention times (Table 2). The gradual sharpening of elution profiles with increasing TBuAA concentration is apparent for all injection volumes, with the most significant effect being noted for the 32-μL injection. The peak width at baseline decreases from about 10 min at 10 mM TBuAA to approximately 5.6 min at 20 mM and 4.3 min at 30 mM. At 30 mM, all injection volumes give Langmuirian elution profiles, but at 10 mM, there is a significant fronting, likely due to the insufficient amount of ion-pairing reagent in the eluent. Similar observations have been made for peptides [23]. As a conclusion, it is necessary to use at least a one-to-one concentration of TBuAA to maintain sharp elution profiles, i.e., to resolve T16 from, for example, earlier-eluting impurities. It is likely that even higher TBuAA concentrations would further improve the elution profile and is a matter that merits further investigation.

**Fig. 5:**
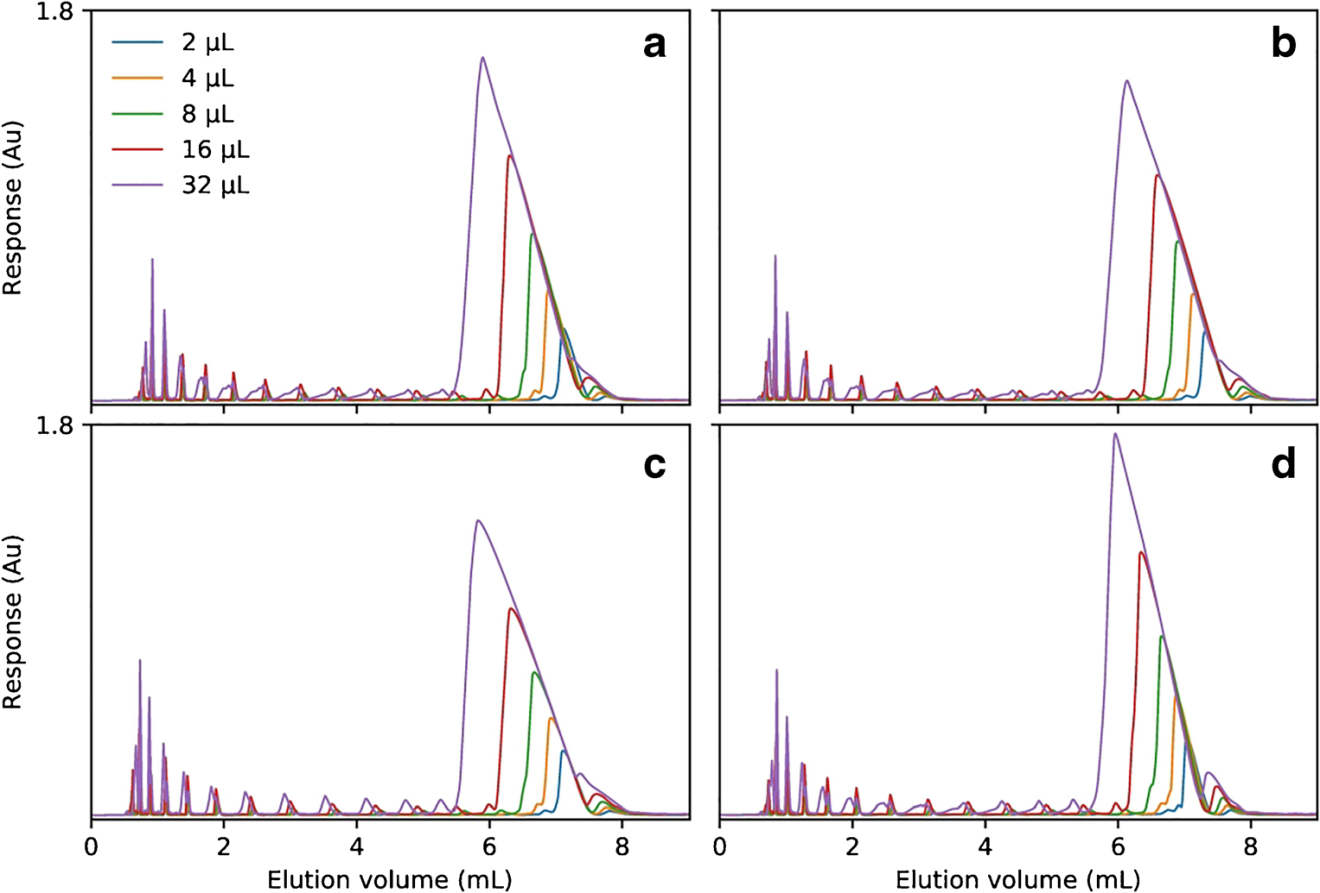
Overloaded elution profiles of preparative injections of 2–32 μL of 9.38 mg mL^−1^ PS-modified T16 on the C4 (**a**), C8 (**b**), C18 (**c**), and phenyl (**d**) columns eluted using 30 mM TBuAA. Gradient start adjusted to normalize the retention volume; gradient slope, 1.14 v% mL min^−1^. See Table 2 for method details

Corresponding experiments were then performed on all columns and the results are presented in Fig. 5. Gradient starting conditions were adjusted so that the retention volume for T16 was approximately 7 mL on all columns (Table 2). This normalization was justified, as the productivity of a preparative method is dependent on the cycle time. The initial gradient adjustments correlate well with the previous analytical results of T5–T20 (Fig. 1d), i.e., the retention was higher on all alkyl-based phases, so the starting amount of acetonitrile needed to be higher than on the phenyl column.

The general results indicate that all columns perform similarly, all having well-defined Langmuir-shaped elution profiles with overlapping tails and well-defined fronts. The peak width at the baseline of the 32-μL injection elution profile revealed that the phenyl column has the narrowest peak width of approximately 1.8 mL. All other columns have a peak width of approximately 2.1 mL. The sharper elution profiles obtained on the phenyl column are also indicated by the height of its profiles.

The trail of smaller peaks eluting before the main peak corresponds to all the shortmers originating from coupling failure during the β-cyanoethyl phosphoramidite solid-phase synthesis [6]. These peaks are nearly symmetrical up to the 32-μL injection volume, where the peaks start to display severe fronting on all columns except C18. On the other hand, the later-eluting impurities are best resolved on the phenyl column.

The overlaid chromatograms of the 2- and 32-μL injections compared with the analytical retention times of T12, T16, T17, and T20 are presented in Electronic Supplementary Material (ESM) Fig. S1 for the C18 and phenyl columns. ESM Fig. S1 indicates that T13–T15 are well separated from T16 on both C18 and phenyl columns with 2-μL injections but that they are probably co-eluting in the front with a 32-μL injection. The later-eluting component is better resolved from the tail of the main peak on the phenyl column. It is noticed that an additional impurity is partially resolved on this column in the front of the main peak at the 2-μL injection.

The stationary phase particles used in this study (2.5 μm particles) are in a size between modern HPLC and UHPLC and could be used for small-scale purification (mg scale) but are not recommended for large-scale preparative separations due to the high pressure drop and to difficulties packing homogenous beds in large diameter columns [10]. The loss of resolution expected when increasing particle size can be compensated by increasing the length of the column to maintain a constant column length over particle diameter ratio. With regards to effects of changing particle size on productivity, each separation problem needs to be individually optimized depending on the constraints (pressure, flow, and column dimensions) of the system [22, 24, 25]. Maintaining a constant particle size squared over column length ratio is a good rule of thumb applicable for binary and isocratic separation problems [10], but its applicability for gradient separation needs to be investigated.

To summarize, according to the elution profiles, the C4, C8, C18, and phenyl columns can be used for preparative purifications of T16, and the phenyl column displays an additional degree of resolution. This can probably be attributed to the π–π interactions between the thymine nucleobase and the phenyl ring on this phase. Since the different nucleobases are expected to differ in their ability to form π–π interactions [26, 27], mixed nucleobase ONs might display other tendencies in selectivity.

### Fraction analysis of T16 separated on C18 and phenyl columns

In order to determine which column is most suitable for purification, a closer investigation of the relative composition of T16 in different segments of its elution profile is necessary. It is particularly relevant to investigate the possible presence of displacement of earlier-eluting shortmers.

**Fig. 6:**
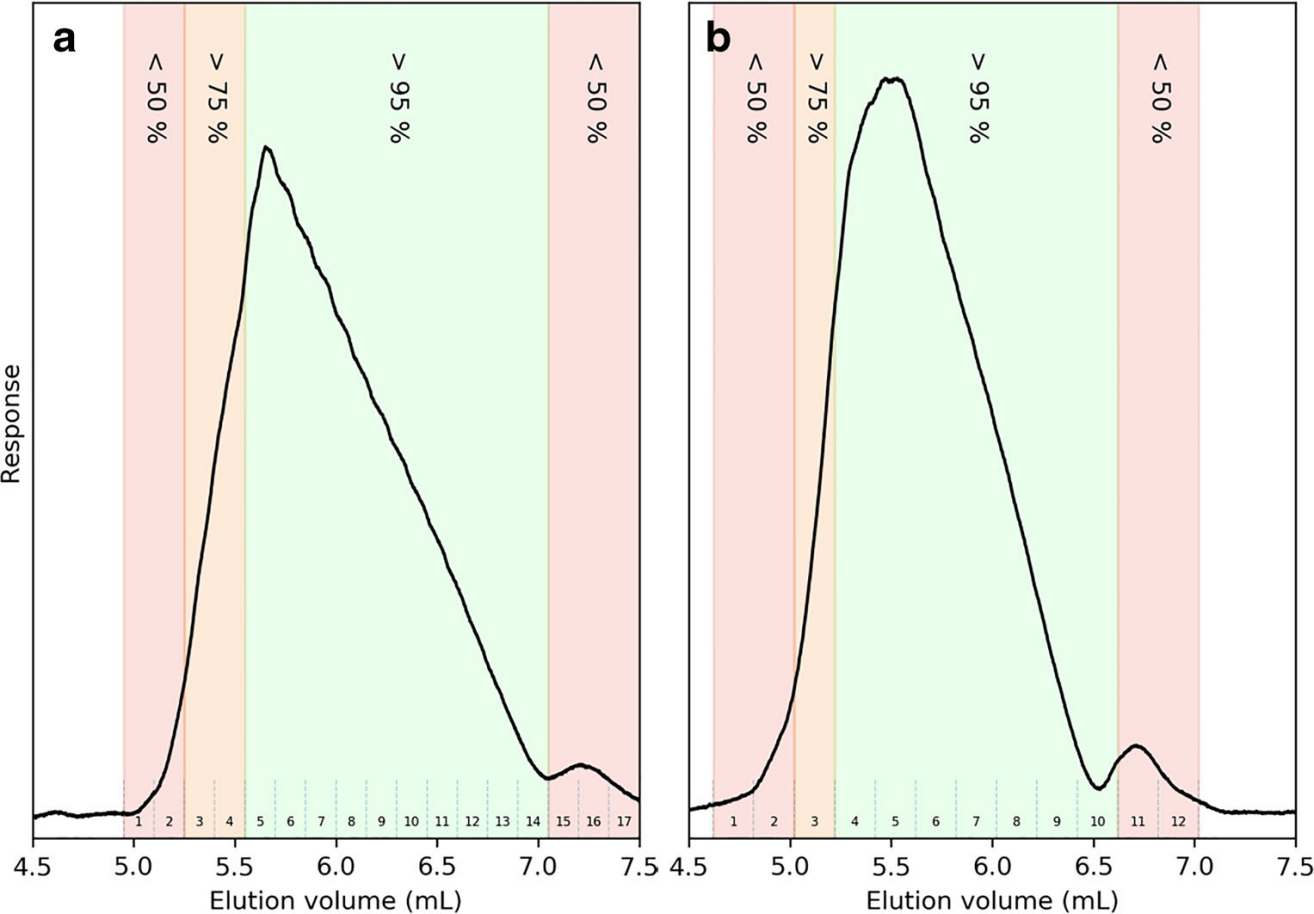
Chromatograms of 10 μL of 30 mg mL^−1^ PS-modified T16 on the C18 (**a**) and phenyl (**b**) columns eluted using 30 mM TBuAA. Dashed numbered vertical lines indicate the collected fractions. The shaded areas indicate the approximate purity of T16 in that area as determined by relative area (UV, response at 300 nm). See Table 2 for method details

Fraction collection and subsequent fraction analysis was therefore performed on the C18 and phenyl columns. The method (Table 2) developed on the Agilent 1200 system was transferred without modification to a Waters ACQUITY I-Class equipped with a fraction collector. With the current system setup, the injection volume was limited to 10 μL, and therefore, the sample concentration was increased by a factor of 3.2 from 9.7 to 31 mg mL^−1^. Mass overloading is often preferred over volume overloading to increase productivity [10]. Detection at 280 nm led to a highly saturated response and had to be adjusted to 300 nm, but it can still be suspected that the response is non-linear (Fig. 6a and b). However, the general characteristics are similar to those obtained on the other system (compare Figs. 5c and d to 6a and b). The widths of the elution profiles at the baseline were measured to be 2.1 mL on the C18 and 1.8 mL on the phenyl column, identical to previous results with the same amount injected, but with a 3.2 times higher injection volume (Fig. 5c and d), indicating no volume overloading.

Fractions were collected every 0.15 mL and 0.2 mL on the C18 and phenyl columns, respectively. The collected fractions are indicated by dashed horizontal lines in Fig. 6. Each fraction was analyzed to evaluate the presence of T13, T14, T15, T16, and T17 (see ESM Table S1). The [M-3H]^–3^ charge state was found to be the most abundant and was therefore extracted and used for further analysis. From the fraction analysis, the relative areas of known components (T13–T17) were calculated and presented in ESM Table S2. Three elution zones where the purity of T16 is less than 50%, greater than 75%, or greater than 95% based on relative area were calculated. These zones are color coded in Fig. 6a and b. The fraction analysis reveals a mixture of shortmers in the front of the elution profiles on both columns. In the tail of the elution profile and in the late-eluting peak, two masses are predominant, one corresponding to T17 and the other likely to the cyanoethyl (CNET) adduct, theoretically adding 53 Da to the total mass of T16 [7, 8]. The theoretical yield in the green (95% purity) zone is 71% for the C18 column and 77% for the phenyl column.

**Fig. 7:**
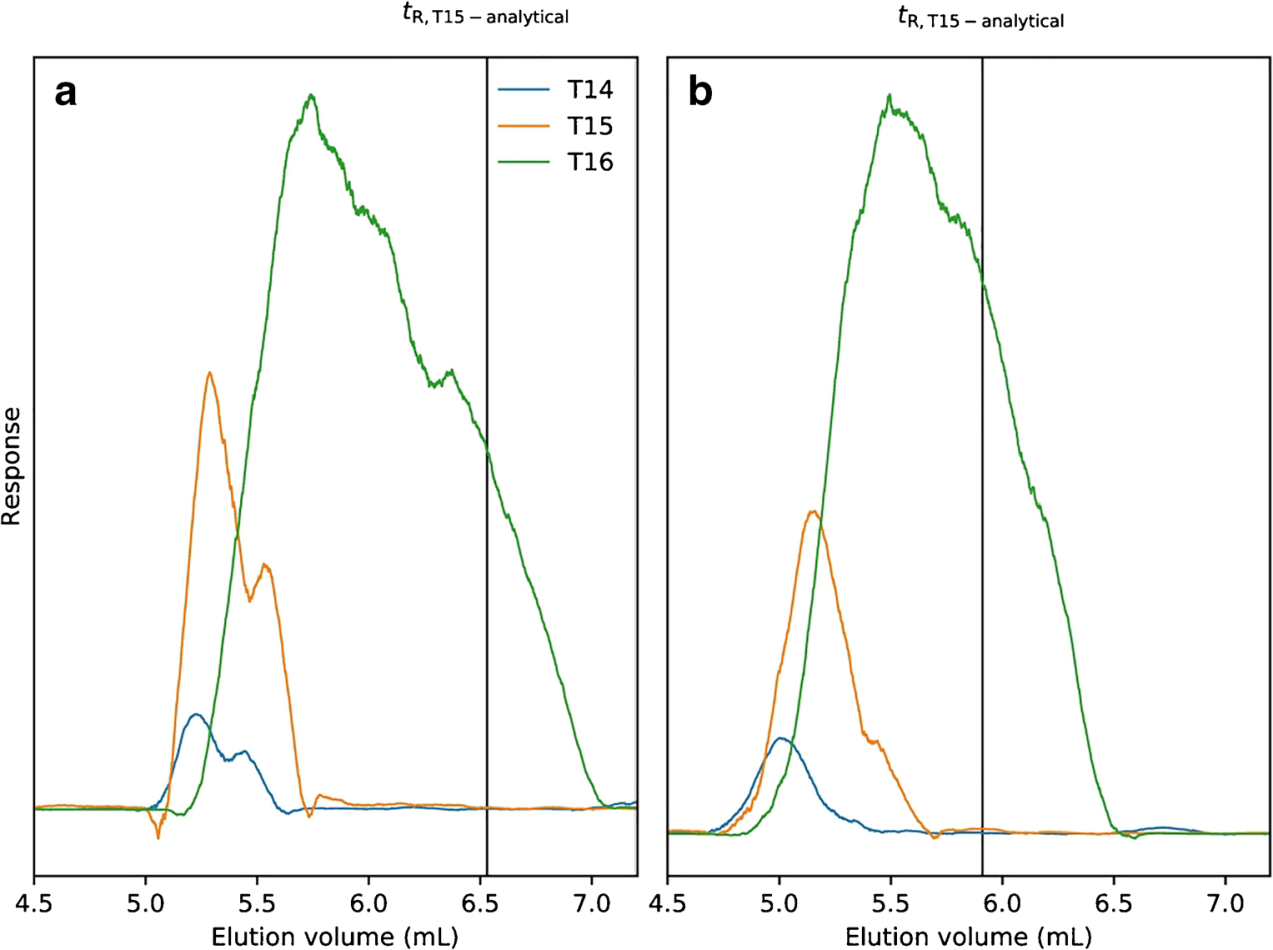
Extracted ion chromatograms of T16 (m/z 1008.12), T15 (m/z 1180.34), and T14 (m/z 1100.19) from 10-μL preparative injections of 30 mg mL^−1^ PS-modified T16 on the phenyl (**a**) and C18 (**b**) columns eluted using 30 mM TBuAA. Vertical line represents the analytical retention volume of T15 when injected separately. See Table 2 for method details. A Savitzky–Golay filter (window size 271, polynomial degree 2) was applied to reduce the noise of the mass spectrometry data. T14 and T15 signals amplified five times for visual purposes

One important and well-characterized phenomenon that can improve productivity in preparative chromatography is the displacement of earlier-eluting components, caused by competition with the high-concentration frontal zone of the target component [28]. To investigate the presence of such displacement, the analytical retention volume of T15 was compared with the peak apex retention volume of T15 from the overloaded T16 injection. The extracted ion chromatograms from the 10-μL injection in Fig. 6a and b are presented in Fig. 7a and b. Data were smoothed using a Savitzky–Golay filter [29]. The most abundant charge state of T14–T16 was found to be [M-4H]^−4^. The displacement of T15 is significant on both columns and explains the broad > 95% purity zone, with high purity (Fig. 6) facilitating an increased yield.

## Conclusions

A fundamental study of ion-pair RPLC for the analysis and purification of both native and PS-modified ONs of lengths between 5mer and 20mer was presented. The reversed-phase chromatography columns C4, C8, C18, and phenyl were systematically compared using two ion-pairing reagents triethylammonium acetate (TEtAA) and tributylammonium acetate (TBuAA), respectively. The following conclusions were made:

(I) There is a large difference in diastereomer selectivity between the columns when using the ion-pair reagent TEtAA. In the analytical investigation, we examined the selectivity of ONs of various lengths, finding that selectivity is dependent on both the ion-pairing reagent and the column chemistry. Using TEtAA, all studied native ONs were resolved. For PS-modified ONs, the diastereomeric selectivity was highest on the C4 and C18 columns, decreasing with increasing ON length.
(II) The phenyl column displayed the highest length-based selectivity for PS-modified ONS using the ion-pair reagent TBuAA. The switch from TEtAA to TBuAA as ion-pairing reagent significantly narrowed the elution profile for PS-modified ONs due to the decreased partial resolution of diastereomers on all columns. The ranking of the columns was found to be phenyl > C18 > C4 ≈ C8. Since most current ASOs are PS-modified, suppression of diastereomer separation is necessary. For a fully modified 20mer containing more than five hundred thousand isomers, separation of those would be impossible. Therefore, regulatory authorities currently do not require the complete or even partial resolution and identification of diastereomers [8]. Hence, all further experiments were performed using TBuAA reagent and fully PS-modified ONs.
(III) The phenyl column displayed better preparative performance than the C18 column due to displacement of shortmers and better separation of later-eluting impurities. In the preparative investigation, the sharpest elution profiles were obtained on the phenyl column, while the three other studied phases displayed similar peak broadening. Finally, to evaluate the separation power of the C18 and phenyl columns, fraction collections and analysis of T16 were performed. T15 was significantly displaced using both types of column materials, facilitating higher purity and yield and consequently lower separation cost for these expensive molecules. In addition, T17 impurities were better separated using the phenyl column, meaning that this column displays slightly better preparative performance than the C18 columns.

### Future work

Available literature about chromatographic purification of PS-modified ONs is still limited and therefore requires fundamental research and a stepwise approach in order to rationally design such purification processes based on, for example, size and sequence of oligonucleotides. The long-term goal is to build a more detailed mechanistic understanding of the ion-pair separation of therapeutic ONs for improved predictions of both analytical and preparative separations. This requires in-depth adsorption and retention mechanism studies and the underlying mechanism of the selected ion-pairing reagents need to be better understood in those chromatographic systems for more complex samples; these areas are currently covered in ongoing studies in our academically and industrial laboratories, respectively.

## Electronic supplementary material

ESM 1 (PDF 528 kb)
