# Supplementary material for: Analytical and preparative separation of phosphorothioated oligonucleotides: columns and ion-pair reagents
Source: Anal Bioanal Chem. 2019 Dec 9;412(2):299–309. doi: 10.1007/s00216-019-02236-9 (PMC6992550; doi:10.1007/s00216-019-02236-9)
Supplement: Supplementary file 1 — (PDF 528 kb) [file 216_2019_2236_MOESM1_ESM.pdf]

Analytical and Bioanalytical Chemistry

Electronic Supplementary Material

Analytical and preparative separation of phosphorothioated oligonucleotides: columns and ion-pair reagents

Martin Enmark, Joakim Bagge, Jörgen Samuelsson, Linda Thunberg, Eivor Örnskov, Hanna Leek, Fredrik Limé, Torgny Fornstedt

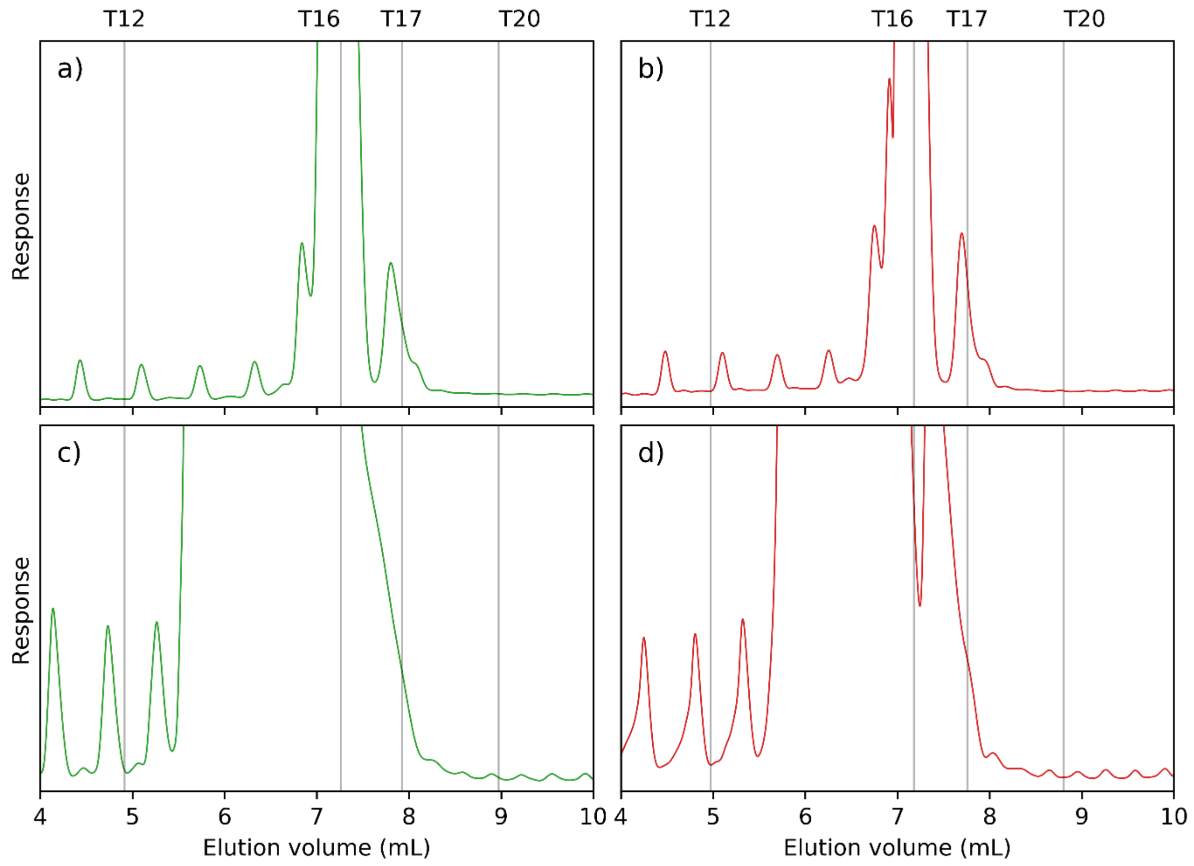

Fig. S1 Chromatograms of injections of T16 on the C18 and phenyl columns eluted using 30 mM TBuAA. Plots (a) and (b) show 2-μL injections on C18 and phenyl columns overlaid with the peak apex retention volumes of analytical injections of T12, T16, T17, and T20 oligos (grey vertical lines). Plots (c) and (d) show 32-μL injections on both columns. See Table 2 (Figs. 5–7) for method details

Table S1 List of the first four possible charge states of fully phosphorothioated deoxythymidine oligonucleotides

		Charge state			
**Oligonucleotide**	**MW (g moL^{-1})**	**[M-1H]$^{-1}$**	**[M-2H]$^{-2}$**	**[M-3H]$^{-3}$**	**[M-4H]$^{-4}$**
T13	4085.3	4084.3	2041.6	1360.7	1020.3
T14	4405.6	4404.6	2201.8	1467.5	1100.4
T15	4725.8	4724.8	2361.9	1574.2	1180.4
T16	5046.1	5045.1	2522.0	1681.0	1260.5
T17	5366.3	5365.3	2682.1	1787.7	1340.5

Table S2 Relative purities of each analysed fraction (fractions 1–17). The white cells contain data from the C18 column and the shaded cells from the phenyl column

Fraction number	Fraction start (mL)		Relative purity (%)											
			T13		T14		T15		T16		T17		Unknowns	
**1**	4.95	4.62	63.2	39.9	36.8	23.9								36.3
**2**	5.1	4.82	19.2	13.0	26.6	18.8	25.1			52.1			29.1	16.1
**3**	5.25	4.92			6.9	5.2	19.6	16.4	73.5	78.4				
**4**	5.4	5.02			2.3		7.7	4.9	90.1	95.1				
**5**	5.55	5.22					3.9		96.1	100.0				
**6**	5.7	5.42							100.0	100.0				
**7**	5.85	5.62							100.0	100.0				
**8**	6	5.82							100.0	100.0				
**9**	6.15	6.02							100.0	100.0				
**10**	6.3	6.22							100.0	68.9				31.1
**11**	6.45	6.42							100.0			11.0		89.0
**12**	6.6	6.62							100.0					100.0
**13**	6.75								100.0					
**14**	6.9								85.8		11.3		2.8	
**15**	7.05										10.6		89.4	
**16**	7.2													
**17**	7.35													
